# Supplementary material for: Splice-Junction-Based Mapping of Alternative Isoforms in the Human Proteome
Source: Cell Rep. Author manuscript; Available in PMC 2020 Jan 15. (PMC6961840; doi:10.1016/j.celrep.2019.11.026)
Supplement: 2 [file NIHMS1546469-supplement-2.zip › DF1/PXD010154/Heart/Heart_65_TTN_INVLEPPK.pdf]

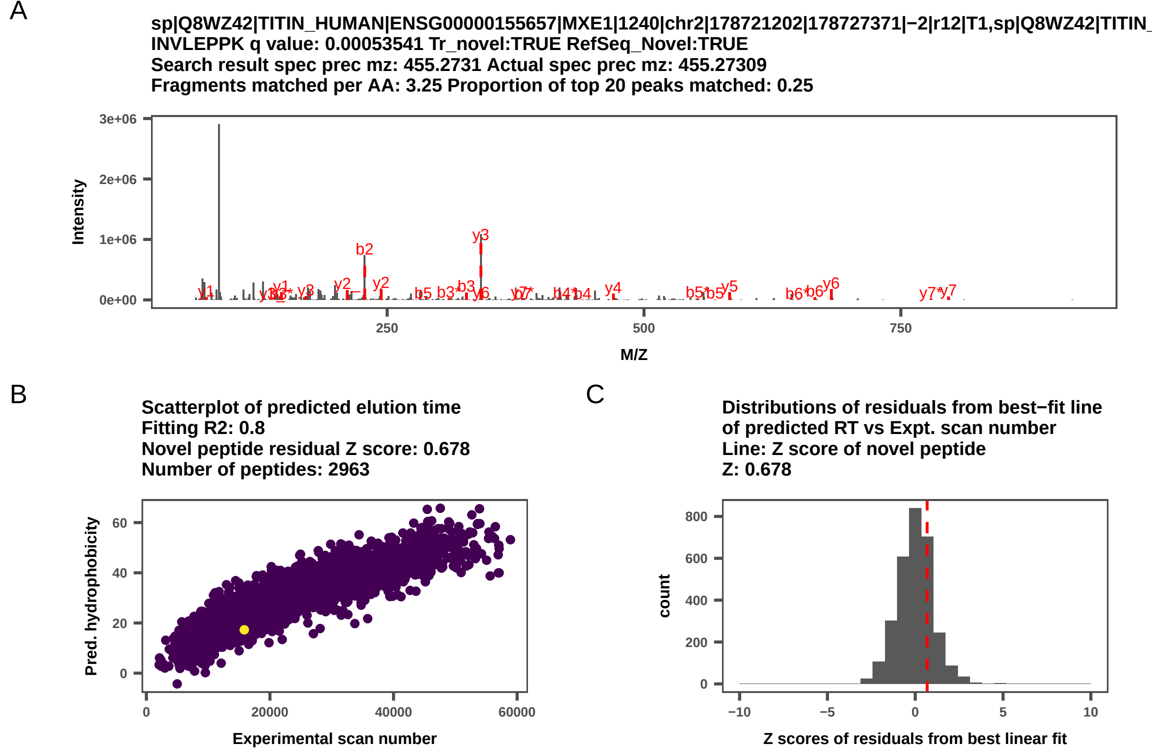
A
sp|Q8WZ42|TITIN_HUMAN|ENSG00000155657|MXE1|1240|chr2|178721202|178727371|−2|r12|T1,sp|Q8WZ42|TITIN_
INVLEPPK q value: 0.00053541 Tr_novel:TRUE RefSeq_Novel:TRUE
Search result spec prec mz: 455.2731 Actual spec prec mz: 455.27309
Fragments matched per AA: 3.25 Proportion of top 20 peaks matched: 0.25
Intensity
3e+06
2e+06
1e+06
0e+00
y3
b2
y2
y2
y1
y4
y5
y6
y7
b5
b3
b6
M/Z
250
500
750
B
Scatterplot of predicted elution time
Fitting R2: 0.8
Novel peptide residual Z score: 0.678
Number of peptides: 2963
Pred. hydrophobicity
60
40
20
0
0
20000
40000
60000
Experimental scan number
C
Distributions of residuals from best−fit line
of predicted RT vs Expt. scan number
Line: Z score of novel peptide
Z: 0.678
count
800
600
400
200
0
−10
−5
0
5
10
Z scores of residuals from best linear fit
